# Informal and paid care for Brazilian older adults (National Health Survey, 2013)

**DOI:** 10.1590/S1518-8787.2017051000013

**Published:** 2017-06-01

**Authors:** Maria Fernanda Lima-Costa, Sérgio Viana Peixoto, Deborah Carvalho Malta, Célia Landmann Szwarcwald, Juliana Vaz de Melo Mambrini

**Affiliations:** I Núcleo de Estudos em Saúde Pública e Envelhecimento. Centro de Pesquisas René Rachou. Fundação Oswaldo Cruz. Belo Horizonte, MG, Brasil; II Departamento de Enfermagem Aplicada. Escola de Enfermagem. Universidade Federal de Minas Gerais. Belo Horizonte, MG, Brasil; III Departamento de Enfermagem Materno Infantil e Saúde Pública. Escola de Enfermagem. Universidade Federal de Minas Gerais. Belo Horizonte, MG, Brasil; IV Instituto de Comunicação e Informação Científica e Tecnológica em Saúde. Fundação Oswaldo Cruz. Rio de Janeiro, RJ, Brasil

**Keywords:** Aged, Caregivers, Disabled Persons, Activities of Daily Living, Socioeconomic Factors, Health Surveys, Idoso, Cuidadores, Pessoas com Deficiência, Atividades da Vida Diária, Fatores Socioeconômicos, Inquéritos Epidemiológicos

## Abstract

**OBJECTIVE:**

To describe the prevalence and sociodemographic factors associated with informal and paid care for Brazilian older adults with functional limitations.

**METHODS:**

Of the 23,815 participants of the National Health Survey aged 60 or older, 5,978 reported needing help to perform activities of daily living and were included in this analysis. The dependent variable was the source of care, categorized as exclusively informal (unpaid), exclusively formal (paid), mixed or none. The socio-demographic variables were age (60-64, 65-74, ≥ 75 years old), gender and number of residents in the household (1, 2, ≥ 3). The multivariate analysis was based on binomial and multinomial logistic regressions.

**RESULTS:**

Informal care predominated (81.8%), followed by paid (5.8%) or mixed (6.8%) and no care (5.7%). The receipt of care from any source increased gradually with the number of residents in a same household, regardless of age and gender (OR = 4.85 and 9.74 for 2 and ≥ 3, respectively). Age was positively associated with receiving any care while the male gender showed a negative association. The number of residents in the household showed the strongest association with informal care (OR = 10.94 for ≥ 3 residents), compared with paid (OR = 5.48) and mixed (OR = 4.16) care.

**CONCLUSIONS:**

Informal care is the main source of help for community-dwelling older adults with functional limitations. In a context of rapid population aging and decline in family size, the results reinforce the need for policies to support long-term care for older Brazilians.

## RESUMO

**OBJETIVO:**

Descrever a prevalência e fatores sociodemográficos associados à ajuda informal e remunerada a idosos com limitações funcionais.

**MÉTODOS:**

Dos 23.815 participantes com 60 anos ou mais da Pesquisa Nacional de Saúde, 5.978 declararam necessitar de ajuda para realizar atividades da vida diária e foram incluídos nesta análise. A variável dependente foi a fonte de ajuda, categorizada como exclusivamente informal (não remunerada), exclusivamente remunerada, mista ou nenhuma. Os fatores sociodemográficos foram idade (60-64, 65-74, ≥ 75 anos), sexo e número de moradores no domicílio (1, 2, ≥ 3). A análise multivariada foi baseada nas regressões logísticas binomial e multinomial.

**RESULTADOS:**

A ajuda informal predominou (81,8%), seguida pela remunerada (5,8%) ou mista (6,8%) e nenhuma (5,7%). A propensão ao recebimento da ajuda por qualquer fonte aumentou gradativamente com o número de moradores no domicílio, independentemente da idade e do sexo (OR = 4,85 e 9,74 para 2 e ≥ 3 moradores, respectivamente). A idade apresentou associação positiva e o sexo masculino apresentou associação negativa com o recebimento de qualquer ajuda. O número de moradores no domicílio apresentou associação mais forte com a ajuda informal (OR = 10,94 para ≥ 3 moradores), em comparação à ajuda remunerada (OR = 5,48) e mista (OR = 4,16).

**CONCLUSÕES:**

O cuidado informal é a principal fonte de ajuda domiciliar aos idosos com limitações funcionais. Em um contexto de rápido envelhecimento populacional e diminuição do tamanho das famílias, os resultados reforçam a necessidade de políticas para garantir o cuidado domiciliar aos idosos com limitações funcionais.

## INTRODUCTION

Aging of the population is the most important demographic change observed around the world in recent decades^a,b^. This demographic change raises concerns about the ability of social systems to meet the growing demand for long-term care due to the increase of functional limitations in the older age groups^1-3^. In different contexts and cultures, care for community dwelling with functional limitations is performed predominantly by informal caregivers (unpaid family and friends), as evidenced by national surveys conducted in North America, Asia and Europe^3-6^ and by an epidemiological study conducted in the city of São Paulo^7^.

The availability of informal care tends to decrease in the near future, as a result of the reduction in the size of families, of the increase in the number of couples without children, and of the increased participation of women in the labor market^3,4^. The economic value of informal care for older adults is rarely considered. Recently, in the United States, the annual cost of informal care for older persons has been estimated in 522 billion dollars, considering the missed income opportunities^8^. The annual cost to replace informal care with non-qualified professionals was estimated at 221 billion dollars and the corresponding cost for qualified professionals was estimated at 642 billion dollars^8^.

In the world context, Brazil is one of the countries where population aging occurs the fastest^a,b^. Brazilians aged 60 years expect to live for over two more decades^c^ and the dependency ratio of older adults (in relation to the potentially productive age segment) has increasing projections^d^. The prevalence of informal and paid care for the older Brazilians is still unknown, since until recently there were no nationwide data on the subject.

In this study, we used data from the National Health Survey^e^ to describe the prevalence and sociodemographic factors associated with informal and paid care for Brazilian older adults with functional limitations who reported needing help to perform activities of daily living.

## METHODS

### Data Source

The National Health Survey (PNS) is a household survey, and its sample represents the adult Brazilian population^9,e^. The survey was conducted in 2013 by the Brazilian Institute of Geography and Statistics (IBGE), in collaboration with the Brazilian Ministry of Health^e^. The survey has three questionnaires: a household questionnaire; an individual questionnaire, to be answered by all residents; and another individual questionnaire, to be answered by a sample of residents aged 18 or older. The module on the functioning of older adults, that generated the information used in this analysis, was addressed to all residents aged 60 or older. For this analysis, the microdata from the survey were obtained in IBGE’s homepage^e^.

### Variables

The module on functioning in the National Health Survey’s questionnaire has separate questions about the degree of difficulty to perform basic and instrumental activities of daily living. Each question has four answer options: no difficulty, little difficulty, much difficulty and cannot do it. For those who reported having any difficulty to perform each activity, it was asked if he/she received help for accomplishing it, with three answer options: yes; no, because there’s no one to help; no, because there is no need for help. For those who reported receiving help, it was asked who offered the help, with seven answer options: unpaid family member residing in the same household; unpaid family member residing in another household; unpaid non-family member; paid family member residing in the same household; paid family member residing in another household; paid professional caregiver; and paid domestic worker.

The functional limitation was attributed to those who reported some degree of difficulty to perform at least one of ten basic or instrumental activities of daily living. The basic activities considered were: feeding, bathing, using the toilet, dressing, walking from one room to another on the same floor and lying down or getting out of bed. The instrumental activities considered were: going shopping, managing one’s own finances, taking medicine and getting out of the house using a means of transport. The need for help to perform activities of daily living was assigned to those who declared receiving help to perform one or more activities and to those who reported needing help, although they did not receive it.

The main analyses of this study were based on the older adults with functional limitations, who reported needing help to carry out one or more activities of daily living. Considering that the older adults may have received help from more than one person to perform the same or different activities, four groups were created: no help (for those who did not receive help to perform any activity, although they reported needing it for one or more activities); exclusively informal care (care provided solely by an unpaid caregiver, family member or not); exclusively formal or paid care (care provided solely by a paid caregiver, family member or not); and mixed care (informal and paid care). In the description of the results, the person who helped the older person to perform at least one activity of daily living was also considered, who was categorized into: unpaid family member residing in the same household, unpaid family member residing in another household, unpaid non-family member, paid caregiver and family member or paid domestic worker.

Other study variables included age (60-64, 65-74, 75 years or older), gender and number of residents in the household (lives alone, resides with one person, resides with two or more persons, corresponding to one, two and three or more residents, respectively).

### Data Analysis

The description of the results was based on means and prevalence, with their respective confidence intervals (95%CI), considering the National Health Survey’s sampling parameters and individual weights^e^. In the unadjusted analyses, the Chi-square and Pearson tests and linear regression were used to examine the statistical significance of the differences between proportions and means, respectively.

The multivariate analyses of sociodemographic factors associated with receiving help were based on binary logistic regression and multinomial logistic regression^10^, estimating the odds ratio and 95%CI. In the first case, the outcome variable was receiving help from any of the aforementioned sources, considering the non-receiving of any help as reference. In the second case, the outcome variable was categorized into exclusively informal help, exclusively paid help, mixed help (informal and paid) and no help, considering the last group as reference. The multivariate models were adjusted by age, gender and number of residents in the household.

Additionally, binomial logistic regression was used to estimate the predicted probabilities of the receiving of any sort of help to perform activities of daily living, by age and number of residents in the household. This model was adjusted by gender and mutually adjusted by the two other variables.

The analyses were carried out using the procedures for complex samples of the statistical package Stata, version 13.0.

### Ethical Aspects

The National Health Survey was approved by the National Ethics Committee for Research with Human Beings (Conep) in June 26, 2013 (Process no. 328.159). All participants signed the informed consent form.

## RESULTS

Among the 23,815 participants of the National Health Survey’s sample aged 60 or older, 7,233 (30.1%; 95%CI 29.2–31.1) reported difficulties to perform one or more activities of daily living. The average age of the participants was 69.9 years, and they were predominantly women (56.4%). Those with functional limitations, in comparison to others, were older (mean = 74.6 years vs. 67.8 years, respectively), in higher proportion of women (64.4% vs. 47.1%) and resided in households with three or more residents (50.4% vs. 49.1%). Among those with functional limitations, 81.2% (n = 5,978) reported receiving or needing help to perform at least one activity of daily living; subsequent analyses were based on those participants (Table 1).

**Table 1:** Sociodemographic characteristics of participants of the sample aged 60 or older, according to their report of having some functional limitation. National Health Survey, 2013.

Characteristic	Total sample		Without limitation		With limitation^b^		p^c^
			
	%^a^	95%CI	%^a^	95%CI	%^a^	95%CI	95%CI
Average age (years)	69.9	69.7–70.1	67.8	67.6–68.0	74.6	74.3–75.0	< 0.001
Age group (years)							
60–64	32.2	31.1–33.2	39.0	37.7–40.3	16.4	15.1–17.8	< 0.001
65–74	41.9	40.8–43.0	44.7	43.5–46.0	35.8	33.4–37.3	
≥ 75	26.0	25.0–27.0	16.3	15.4–17.3	48.3	46.3–50.3	
Gender							
Female	56.4	55.6–57.2	47.1	46.1–48.0	64.4	62.8–66.0	< 0.001
Male	43.6	42.8–44.4	52.9	52.0–53.9	35.6	34.0–37.2	
Number of residents in the household							
1	14.9	14.2–15.7	14.4	13.6–15.3	16.0	14.7–17.4	0.017
2	35.6	34.4–36.8	36.5	35.1–37.9	33.5	31.5–35.6	
≥ 3	49.5	48.2–50.8	49.1	47.6–50.6	50.4	48.3–52.5	
Needing help to perform at least one activity	-		-		81.2	79.4–82.8	
Number of respondents	23,815		16,582		7,233		

^a^ % (or average, when specified), weighted by the sampling parameters.

^b^ Having at least some difficult in feeding, bathing, using the toilet, dressing, walking on the same floor, lying down or getting out of bed, going shopping, managing money, taking medicine or using a means of transport.

^c^ For differences between those with and without limitation (linear regression for differences between means and Pearson’s Chi-square test for differences between frequencies).

As described in Table 2, among the older adults who reported needing help to carry out one or more activities of daily living, 5.7% did not receive any help, 81.8% received exclusively informal help, 5.8% received exclusively paid help and 6.8% received mixed help. Among those who declared receiving help, 62.0% received help from an unpaid family member who resided in the same household as them, 35.8% from an unpaid family member who resided in another household, 4.9% from an unpaid caregiver, family member or not, 3.4% from paid professional caregivers and 10.3% from a paid family member or domestic worker.

**Table 2:** Sources of help to perform at least one activity of daily living among those who reported receiving or needing help to carry it (them) out. National Health Survey, 2013.

Source of help	%^a^	95%CI
Source of care among those who received or needed it (n = 5,978)^b^		
Did not receive help though it was needed	5.7	4.8–6.7
Received informal help only	81.8	80.1–83.4
Received paid help only	5.8	4.8–6.8
Received both informal and paid help	6.8	5.7–8.0
Person who helped them to perform at least one of the activities (n = 5,619)^b^		
Unpaid family member residing in the household	62.0	59.9–64.2
Unpaid family member not residing in the household	35.8	33.7–38.0
Unpaid non-family member	4.9	4.0–6.0
Paid caregiver	3.4	2.7–4.3
Paid family member or domestic worker	10.3	9.0–11.7

^a^ Weighted by the sampling parameters.

^b^ Number of respondents.

Table 3 shows the distributions of the sociodemographic characteristics of the participants of the study, according to the help received. In total, 55.0% of the help (informal, paid or mixed) were provided to those aged 75 or older, 67.0% to women and 53.6% to people residing in households with three or more residents. Compared to those who did not receive any help, those who received informal, paid or mixed care were more likely to be older, women and lived in households with more residents.

**Table 3:** Sociodemographic characteristics of participants of the sample aged 60 or older who needed help to carry out activities of daily livinga, according to the source of help for carrying out these activities. National Health Survey, 2013.

Characteristic	No help		Any help (informal or unpaid)		Source of help					

					Informal only		Paid only		Informal and paid	
				
	%^b^	95%CI^b^	%^b^	95%CI^b^	%^b^	95%CI^b^	%^b^	95%CI^b^	%^b^	95%CI^b^
Age group (years)										
60–64	26.2	20.2–33.2	13.5	12.1–15.0	13.9	12.4–15.5	14.5	9.9–20.7	7.8	4.5–13.2
65–74	48.0	40.2–56.0	31.5	29.5–33.6	32.4	30.3–34.7	28.6	22.1–36.2	22.7	16.6–30.4
≥ 75	25.8	10.8–32.8	55.0	52.8–57.1	53.7	51.4–55.9	56.9	48.7–64.7	69.5	61.1–76.8
							p^c^ < 0.001			
Gender										
Female	53.8	46.5–61.0	67.0	65.2–68.8	66.9	64.9–68.8	66.8	58.8–73.2	69.2	61.6–38.4
Male	46.2	39.1–53.5	33.0	31.2–34.8	33.1	31.3–35.1	33.2	26.8–40.2	30.8	24.2–38.4
					p^c^ = 0.006					
Number of residents in the household										
1	48.3	40.6–56.1	13.9	12.5–15.5	12.7	11.1–14.2	22.5	16.5–29.8	23.2	17.1–30.7
2	28.5	22.3–35.6	32.5	30.2–34.8	32.2	29.8–34.6	27.7	21.5–34.9	40.3	32.4–48.6
≥ 3	23.1	17.4–30.2	53.6	51.2–56.0	55.3	52.8–57.8	49.9	42.1–57.6	36.6	28.9–45.0
							p^c^ < 0.001			
Number of respondents	359				4,890		383		346	

^a^ Having at least some difficult to perform one of the following activities: feeding, bathing, using the toilet, dressing, walking on the same floor, lying down or getting out of bed, going shopping, managing money, taking medicine or using a means of transport.

^b^ Prevalence and confidence interval (95%CI) weighted by the sampling parameters.

^c^ Pearson’s Chi-square test for difference between the groups.

The results of the multivariate analysis of the socio-demographic factors associated with the help received can be found in Table 4. Older age (75 years) showed positive and statistically significant association (OR = 5.18) with receiving any sort of help, while the male gender showed inverse association (OR = 0.53). The propensity to receiving any sort of help gradually increased with the number of residents at the household (OR = 4.85 and OR = 9.74 for two and three or more residents, respectively). The directions (positive or negative) of the aforementioned associations were consistent for different sources of help. However, some differences in the magnitudes of these associations were observed. Age showed stronger association with receiving mixed help (OR = 10.68) compared to exclusively informal help (OR = 4.88) and exclusively paid help (OR = 4.65). The number of residents at the household had stronger association with receiving exclusively informal help (OR = 10.94 for households with three or more residents) in comparison to exclusively paid or mixed help (OR = 5.48 and OR = 4.16, respectively).

**Table 4:** Results of the multivariate analysis of sociodemographic factors associated with the source of care for older adults with functional limitations who needed help to carry out activities of daily livinga. National Health Survey, 2013.

Characteristic	Any help (informal or unpaid)		Source of help					

			Informal only		Paid only		Informal and paid	
			
	OR^b^	95%CI^b^	OR^c^	95%CI^c^	OR^c^	95%CI^c^	OR^c^	95%CI^c^
Age in years (vs. 60−64)								
65–74	1.51	0.98–2.33	1.52	0.98–2.35	1.22	0.66–2.28	1.83	0.81–4.11
≥ 75	5.18	3.20–8.39	4.88	3.01–7.92	4.65	2.40–9.00	10.68	4.67–24.40
Male (vs. female)	0.53	0.38–0.73	0.53	0.38–0.73	0.55	0.35–0.87	0.50	0.31–0.82
Number of residents in the household (versus 1)						
2	4.85	3.13–7.49	5.27	3.39–8.20	2.52	1.36–4.67	3.80	2.06–7.01
≥ 3	9.74	6.24–15.23	10.94	7.00–17.12	5.48	2.92–10.30	4.16	2.21–7.80

^a^ Feeding, bathing, using the toilet, dressing, walking on the same floor, lying down or getting out of bed, going shopping, managing money, taking medicine or using a means of transport.

^b^ OR (95%CI): Odds ratio (with a 95% confidence interval) mutually adjusted for all variables listed in the table and estimated by logistic regression, with the group that did not receive help as reference.

^c^ OR (95%CI): Odds ratio (with a 95% confidence interval) mutually adjusted for all variables listed in the table and estimated by multinomial logistic regression, with the group that did not receive help as reference.

The Figure shows the predicted probability of receiving any sort of help (informal, paid and/or mixed) to perform activities of daily living, according to age and the number of residents at the household. Compared to those who did not receive help, the likelihood of receiving help was clearly lower among people who lived alone, compared to those residing in households with two and three or more residents. With the increase in age, the likelihood of receiving help of people who lived alone also increased.

**Figure:**
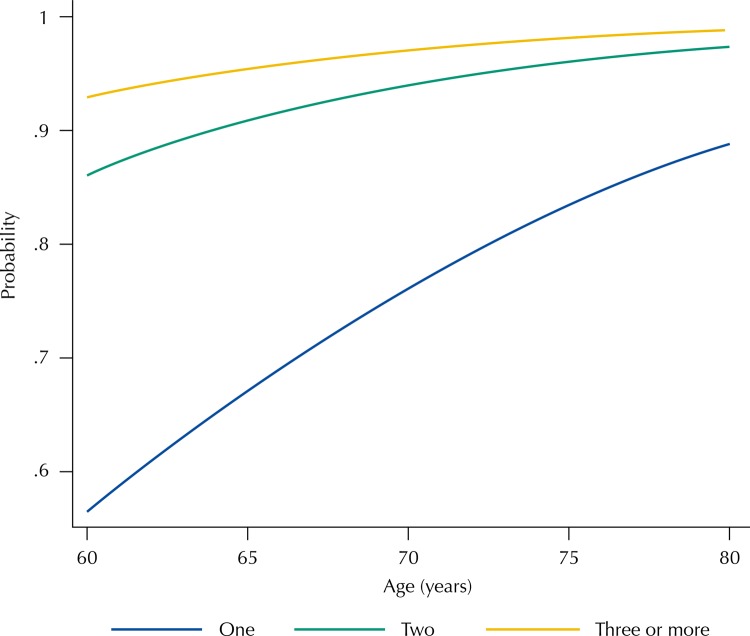
Predicted probabilitya of receiving any sort of helpb to perform activities of daily living among older adults with functional limitations that needed help to carry out these activities, according to age and number of residents in the household. National Health Survey, 2013.

^a^ Probabilities estimatsion and adjusted by age, gender and number of residents in the household.

^b^ Feeding, bathing, using the toilet, dressing, walking on the same floor, lying down or getting out of bed, going shopping, managing money, taking medicine or using a means of transport.

## DISCUSSION

The main results of this study, which was based on a representative sample of Brazilian older adults, are: about 1/3 had difficulties to perform activities of daily living and, of these, 81% reported needing help to perform one or more of these activities; among those who needed help, exclusively informal care prevailed; the propensity to the receiving of help to perform activities of daily living gradually increased with the number of residents in the household, regardless of gender and age.

The meeting of the demand for the care for older adults is a growing concern in different societies^1,3^. However, there are only a few international comparisons concerning the care for older adults, based on nationally representative samples^4,6^. In a recent publication, the prevalence of informal and paid care for older adults in Spain, the United States and England was compared^6^. Despite having some methodological differences in relation to the National Health Survey, the information resulted from those investigations are useful as parameter of comparison to the results of this analysis, as shown below.

The prevalence of the difficulty to perform one or more activities of daily living, in the population aged 50 or older, was of about 25% in Spain and England and 40% in the United States^6^. The corresponding prevalence for Brazilians aged 60 or older is within this range. Among those with functional limitations, the proportion of those who received help to perform activities of daily living was lower in the United States (39%) compared to Spain (75%) and England (67%)^6^. In Brazil, more than 90% of older adults received help to perform one or more of these activities, but it is important to stress that the Brazilian information is restricted to participants who reported needing help to carry out those activities, while in the aforementioned countries, the information refers to all participants with functional limitations, without restriction regarding their perception of the need for help to perform such activities.

Informal care to older adults predominates in different countries, but its prevalence and composition can vary, depending on the existence of policies to support such care, on socioeconomic status, on family arrangements and on cultural factors, among others^1,3-6^. In Spain, England and the United States, between 55% and 64% of older persons with functional limitations receive informal care from residents in the same household^6^. The corresponding prevalence for Brazil was found to be within this range. On the other hand, the prevalence of informal care by persons residing in another household was higher in Brazil (36%) than in Spain (6%) or in England and the United States (about 25% in both)^6^. The prevalence of paid care in Brazil (14%) was similar to that observed in Spain (12%) and higher than in the United States (2%) and in England (5%)^6^. Still in what concerns paid care, it is important to highlight that, in Brazil, paid care provided by a relative or paid domestic worker predominated, to the detriment of paid professional caregivers.

The household arrangements are associated with the source of care for older persons in different societies and cultures, with higher prevalence of informal care in bigger families^4,6^. Our results are in line with these observations. The number of residents in the household showed strong association with receiving help to perform activities of daily living. This association was consistently observed for the different sources of care (informal, paid or mixed), but the magnitude of the association was greater for informal care. It is important to stress that the probability of receiving help by any source was markedly lower among older adults who lived alone. Due to the cross-sectional nature of this study, it is not possible to know if these family arrangements preceded or were a consequence of the functional limitation, leading the older adult to move into a household with more residents. Independently of that, our results reinforce the concerns about the future availability of informal care^3,4^, as a result of the reduction in the size of families and an increase in the number of childless couples, which are significant recent demographic changes in Brazil^11,12^.

The prevalence of functional limitation in Brazilian older adults was higher among women than among men, confirming observations concerning different populations^13^. In contrast, men with need for help to perform activities of daily living were less likely to receive both informal and paid or mixed care, regardless of other demographic characteristics. The predominance of care for women in relation to men is in line with what has been observed in other countries^13^ and future studies are needed for a better understanding of the differences between genders.

This study has advantages and limitations. The main advantage is the large population, with national representation. This allowed, for the first time, ascertaining the magnitude and some factors associated with informal and paid care for Brazilian older adults. Another advantage of the study is its internal validity, since the National Health Survey produced good quality data, with careful elaboration of the instruments and control of the quality of the information^e^. The spouse and the children are important sources of informal care to older adults^4,7,13,14^. Based on the data from the National Health Survey, it is not possible to establish the family relationship of the informal caregiver with the older adult, being this one of the limitations of this analysis. Other limitations are those inherent to the cross-sectional nature of this research, as previously stated.

In 2006, the Ministry of Health established the National Health Policy for Older Adults, stating that it is essential to consider functional condition in their actions^15^, bringing a new paradigm to the discussion on the topic. But there are still no national policies geared towards the support to long-term home care for older adults with functional limitations. Brazil has the fifth largest population in the world, with about 26,500,000 persons aged 60 or older^c^. Applying the results of this analysis to the population in this age group, it is estimated that approximately 6,500,000 of older Brazilians need help to perform activities of daily living, 360,000 do not receive this aid, though they need it, and at least 5.7 million of family members or friends are involved in unpaid care for older adults. These numbers help visualize the dimension of the challenge to be faced by the Brazilian society to ensure the long-term care for older adults with functional limitations.

## Footnotes

a National Institute on Aging; World Health Organization*.* Global health and aging Washington, DC: National Institutes of Health; 2011. (NIH Publication, Nº. 11-7737).

b United Nations, Department of Economic and Social Affairs, Population Division*.* World population prospects: the 2010 revision. New York; 2010 [cited 2015 Dec 3]. Available from: http://esa.un.org/unpd/wpp/index.htm

c Instituto Brasileiro de Geografia e Estatística. Resultados do censo de 2010. Rio de Janeiro: IBGE; 2010 [cited 2015 Oct 15]. Available from: http://www.ibge.gov.br/home/estatistica/populacao/censo2010

d United Nations, Department of Economic and Social Affairs, Population Division. World population prospects: the 2015 revision. New York; 2015 [cited 2015 Dec 3]. Available from: https://esa.un.org/unpd/wpp/publications/files/key_findings_wpp_2015.pdf

e Instituto Brasileiro de Geografia e Estatística. Pesquisa Nacional de Saúde 2013: percepção do estado de saúde, estilos de vida e doenças crônicas. Brasil, grandes regiões e unidades da federação. Rio de Janeiro: IBGE; 2013 [cited 2015 Oct 15]. Available from: www.ibge.gov.br/home/estatistica/populacao/pns/2013.
